# Exploring the functional microbiome of pigs within the porcine respiratory disease complex: viral-bacterial co-infections and virulence factor profiling

**DOI:** 10.1128/spectrum.01910-25

**Published:** 2026-01-29

**Authors:** Adelaide Panattoni, Ilke De Boeck, Stijn Wittouck, Pauline Deffner, Kathrin Lillie-Jaschniski, Julia Stadler, Sarah Lebeer, Sebastiaan Theuns

**Affiliations:** 1 Laboratory of Applied Microbiology and Biotechnology, Department of Bioscience Engineering, University of Antwerp, Antwerpe, Belgium; 2 PathoSense BV 26660, Gent, Belgium; 3 Clinic for Swine, Centre for Clinical Veterinary Medicine, Ludwig-Maximilians-Universität München, Oberschleissheim, Germany; 4 Ceva Santé Animale, Düsseldorf, Germany; University of Arkansas Fayetteville, Fayetteville, Arkansas, USA

**Keywords:** respiratory microbiome, low-biomass samples, finisher pigs, virulence factors

## Abstract

Respiratory infections are among the most impacting on pigs’ health and economic productivity. Despite this, detailed insights into the microbial community of the lower respiratory tract (LRT) are currently lacking, mainly because of difficulties in the processing of respiratory samples. In this study, we characterized the microbiota of the LRT of finisher pigs aged 3–5 months with respiratory symptoms for both the viral and bacterial components, using a previously validated metagenomic diagnostic assay and a full-length 16S rRNA gene sequencing approach, respectively. Functional characterization was carried out using metagenomic shotgun sequencing, revealing the presence of specific virulence factors (VFs). Porcine Reproductive and Respiratory Syndrome Virus (PRRSV) and swine Influenza A Virus (swIAV) were the most prevalent viruses, being detected in 30% and 23% of the tested samples, respectively. *Mesomycoplasma hyopneumoniae*, *Glaesserella parasuis*, and *Pasteurella multocida* were the three most abundant bacterial taxa based on both sequencing approaches, while other detected bacterial taxa consisted mainly of *Streptococcus*, *Clostridium*, and *Rothia* species. Detected virulence factors belonged mainly to *Mesomycoplasma* and *Pasteurella* and consisted of adhesion factors such as *p102*, *p97*, *p146*, *mhp108, mhp107* and the hemolysin-encoding gene *hly*A for *Mesomycoplasma*, and adhesin-encoding *ptf*A and endoxtoxin-related gene *lpxC* for *Pasteurella*. Our data show how the microbial community of the lower respiratory tract in pigs with respiratory symptoms includes key viral (PRRSV, swIAV) and bacterial pathogens (*M. hyopneumoniae*, *G. parasuis*, and *P. multocida*), along with specific virulence factors likely contributing to disease.

**IMPORTANCE:**

The obtained results offer insights into the composition of the swine respiratory tract microflora, opening new perspectives on its correlation with viral infections, functional characteristics, and overall health conditions. Moreover, the present study provides technical advancement on the possibility of extracting and amplifying bacterial DNA from low-biomass respiratory samples, with the resulting possibility of identifying virulence factors and better understanding their contribution to the disease state. These discoveries pave the way for future studies aimed at improving diagnostic accuracy and treatment strategies for respiratory disease in both veterinary and human medicine.

## INTRODUCTION

Respiratory infections in pigs, caused by both viruses and bacteria, constitute a serious issue that concerns the animals’ overall health, the increased necessity of treatments (e.g., antibiotics), their reduced growth performance, and the resulting economic loss (1). Respiratory disease in pigs has also been termed as Porcine Respiratory Disease Complex (PRDC) since multiple pathogens can be present at the same time (2). Among the main known pathogens that contribute to PRDC are Porcine Reproductive and Respiratory Syndrome virus (PRRSV), swine Influenza A Virus (swIAV), as well as bacteria such as *M. hyopneumoniae*, *Bordetella bronchiseptica*, *P. multocida*, *Actinobacillus pleuropneumoniae*, *Streptococcus suis*, and *G. parasuis* (3). Recent metagenomics studies have also shown the presence of other neglected and/or novel viruses (e.g., porcine respirovirus, porcine cytomegalovirus, porcine hemagglutinating encephalomyelitis virus) and bacteria (e.g., *Moraxella* sp., *Rothia* sp.) as part of the respiratory tract community in diseased animals (4), whose contribution to the disease is still uncertain.

Increasing evidence suggests that the endogenous microbes and their relative abundances can contribute to or hinder the onset of diseases depending on how they interact with each other and the host (5). Despite this information having been gathered mainly in the enteric environment, this also applies to the respiratory microbiota, which plays an important gatekeeper function in the airways, with the potential to impact the animal’s overall health (6, 7). In this complex scenario, it is of key importance to obtain more information about the microbial composition of the airways in pigs, from both a compositional and functional point of view.

A few studies have yielded varying results regarding the average composition of the pig respiratory microbiome in both the upper (URT) and lower (LRT) respiratory tract. For instance, one study carried out in Spain and the United Kingdom on 100 piglets using partial 16S rRNA gene (V3-V4) sequencing showed that healthy or asymptomatic animals mainly presented *Moraxella* and *Weeksella* genera in the URT (8). Others in the United States found *Pasteurellaceae* as the dominating family in the tonsils of 12 healthy pigs analyzed by pyrosequencing of V4 region, including varying relative abundances of *Actinobacillus*, *Haemophilus,* and *Pasteurella* (9). As far as the LRT is concerned, the currently available literature is scarce, and to the best of our knowledge, only a few studies investigated the LRT microbiota composition of healthy pigs, yielding conflicting results, as reviewed (6). For instance, *Prevotella* and *Lactobacillus* were identified as main genera in the lungs of 7 healthy pigs from China analyzed by V3-V4 region amplification (10), whereas *Lactococcus*, *Enterococcus*, *Staphylococcus,* and *Lactobacillus* were found in eight healthy animals analyzed via V3-V4 sequencing approach in China (11). Differences in experimental design, geographical location, sampling strategies, and sequencing techniques throughout the different studies could explain these discrepancies (12). An important limitation of these studies is also the use of partial 16S rRNA amplicon sequencing (mostly V3-V4 region), not allowing accurate sub-genus classification up to species level.

In addition to the studies in healthy animals, few studies analyzed the LRT microbial composition in animals showing respiratory clinical signs. For example, *Glaesserella parasuis* and *Mesomycoplasma hyorhinis* have been detected in the lungs of 10 Chinese PRRSV-infected pigs (13), while genera *Haemophilus*, *Pasteurella*, and *Bordetella* have been detected in the LRT microbiota of 20 pigs with respiratory symptoms (e.g., cough, fever, joint swelling, wheezing) in another Chinese study (11). In both cases, microbiome analysis was performed through V3-V4 region sequencing. Furthermore, the genera *Mesomycoplasma*, *Ureaplasma*, and *Haemophilus* have been detected as dominant in seven pig lungs with severe lesions from China through V3-V4 sequencing (10). Despite these studies providing a list of bacteria that seem to be associated with respiratory symptoms, finding an association between microbial composition and disease is particularly complex, considering how many of the aforementioned bacteria involved with PRDC are also present in the respiratory environment in a healthy state, such as *S. suis* and *G. parasuis* (14–17).

To better understand the health and disease dynamics, especially for microorganisms that can be both commensal and pathogens, it is of key importance to uncover not only the microbial composition related to a (sub)clinical state but also the functional characteristics of both microbial flora and pathogens, for example, the virulence factors that are encoded. This knowledge could enhance our comprehension of health and disease states in pigs, leading to improved and more sustainable animal production. In the present study, we performed an extensive microbial characterization of the LRT through tracheo-bronchial swabs collected from 225 finisher pigs with acute respiratory disorders in Germany using innovative full-length 16S rRNA gene sequencing, shotgun metagenomics and a commercially available metagenomics-based diagnostics assay. The integrated approach of state-of-the-art sequencing and diagnostic techniques allowed the detection of viruses, determination of the bacterial composition, and analysis of a known virulence factors panel. The primary aim of this work was to provide a proof-of-concept for this comprehensive methodology and to explore the detailed microbial and functional results obtained from these challenging samples.

## RESULTS

### Composition of the LRT microbiome is dominated by a few bacterial taxa, independent of sequencing technique

**Fig 1:**
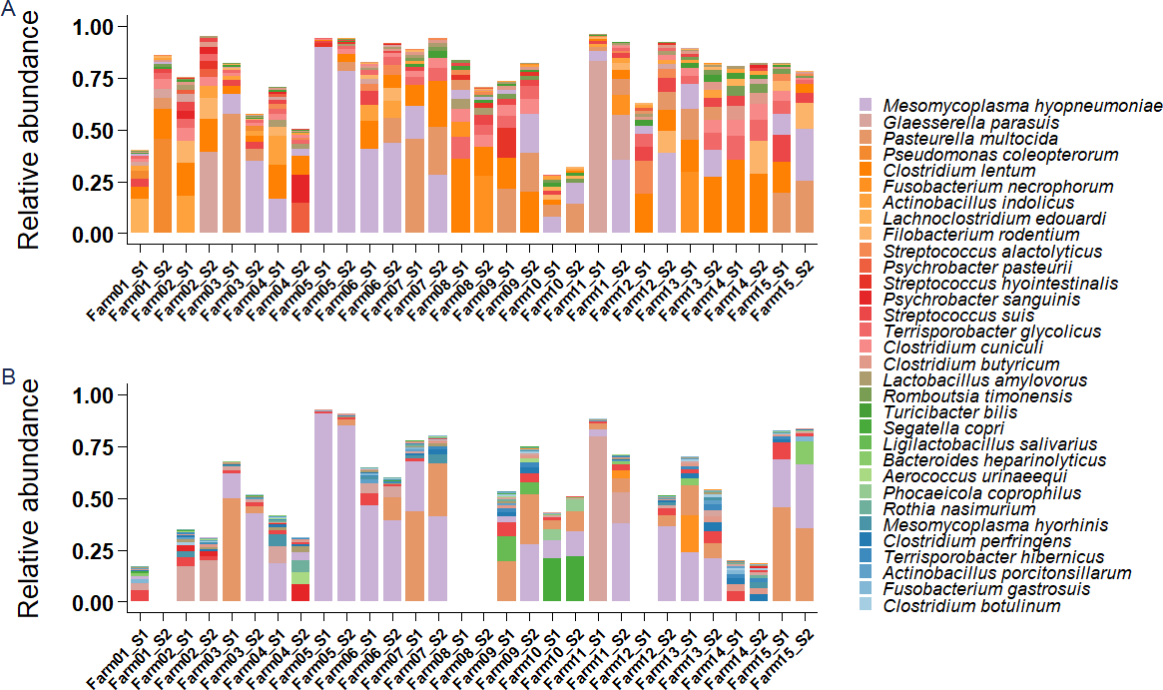
(**A**) Bacterial relative abundances of 20 most abundant bacterial species detected with full-length 16S rRNA gene sequencing. (**B**) Relative abundance of bacterial species detected through metagenomic shotgun sequencing. Empty columns correspond to samples that have not been sequenced using bacterial shotgun metagenomics but only 16S-based sequencing, as insufficient sample volume remained for shotgun metagenomic processing.

Within this study, 15 individual tracheobronchial swabs (TBS) were collected from each one of the 15 farms, generating a total of 225 original samples. These were processed to create two pools per farm, generating a total of 30 pooled samples. These 30 pooled tracheobronchial samples from diseased pigs were then analyzed using 16S rRNA gene sequencing and bacterial metagenomics shotgun sequencing. Bacterial relative abundances obtained with both techniques are represented in Fig. 1A and B. The main (opportunistic pathogens) bacteria found were *M. hyopneumoniae*, *P. multocida*, *G. parasuis*, and *S. suis*, whose mean relative abundances calculated across all farms were 20.95%, 10.93%, 5.97%, and 2.89%, respectively. Other species detected are generally known to be commensals and belonged mainly to genera *Streptococcus* (*S. alactolyticus*, *S. hyointestinalis*), *Clostridium* (*C. lentum*, *C. butyricum*, *C. cuniculi*), *Psychrobacter,* and *Rothia*. For other taxa that were less prevalent, results differed between the two techniques. *Clostridium lentum*, for instance, was only detected through 16S rRNA gene sequencing, while *Bacteroides fragilis* (not in the top 20 most abundant bacteria and therefore not represented in Fig. 1) was only detected with shotgun metagenomic sequencing. Some bacteria were detected in both cases but with different relative abundances. This was the case of *Rothia nasimurium*, *Actinobacillus porcitonsillarum*, and *Bacteroides heparinolyticus*, which were in the 20 most abundant bacteria according to shotgun metagenomics, but only detected in low abundances with 16S rRNA gene sequencing. This discrepancy validates our decision to employ both techniques for the analysis. In some of the farms (e.g., Farms 2, 3, 4, 11), it was possible to observe a discrepancy in microbial composition between the two pools. This observed difference is a logical consequence of the fact that the two pools derive from two different sets of animals within the same farm. In addition, some inter-sample diversity falls within the expected range of technical and biological variability when analyzing distinct microbiome subsamples. Significant variation in microbial composition was observed between farms. The underlying factors contributing to these differences required further investigation.

### Bacterial shotgun metagenomic sequencing highlights the presence of virulence factors in the main opportunistic pathogens

**Fig 2:**
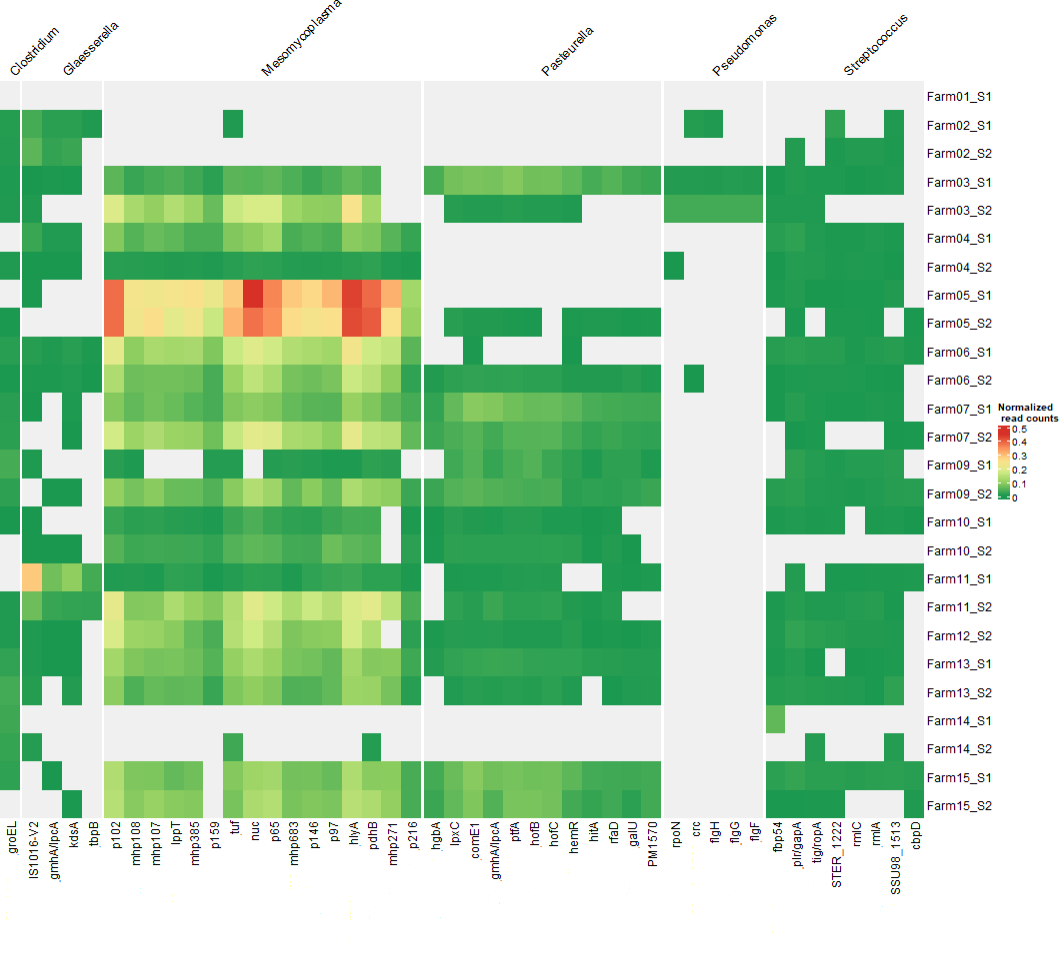
Virulence factors detected in the analyzed TBS samples. Virulence factors that appear at least 10 times in at least two different samples have been kept, while the rest have been filtered out. Data were also normalized based on the total bacterial read number (see Materials and Methods).

While taxonomic evaluation provides an interesting overview, the use of shotgun metagenomic sequencing has the clear added value that it can provide information about functional properties such as virulence factors. Using this technique, we detected an array of virulence factor genes across samples (Fig. 2). The most frequently detected virulence factors belong to the genera *Mesomycoplasma* and *Pasteurella*, followed by genera *Glaesserella*, *Streptococcus*, and *Pseudomonas. Mesomycoplasma* exhibited the highest number of virulence factors, mirroring its consistently high relative abundance in the different samples. This is especially visible in Farm 05, which contains the highest count of *p102*, *p65*, *tuf, nuc, hlyA,* and *pdhB. Glaesserella* was characterized by high relative abundance of *IS1016-V2*, while *Pasteurella* was associated with multiple virulence factors distributed across several samples. Interestingly, *Streptococcus*- and *Pseudomonas*-associated virulence factors were detected at lower levels and in fewer samples, which may indicate their limited presence or lower pathogenic potential in this context.

### Diagnostic results show high numbers of PRRSV and Influenza A virus

**Fig 3:**
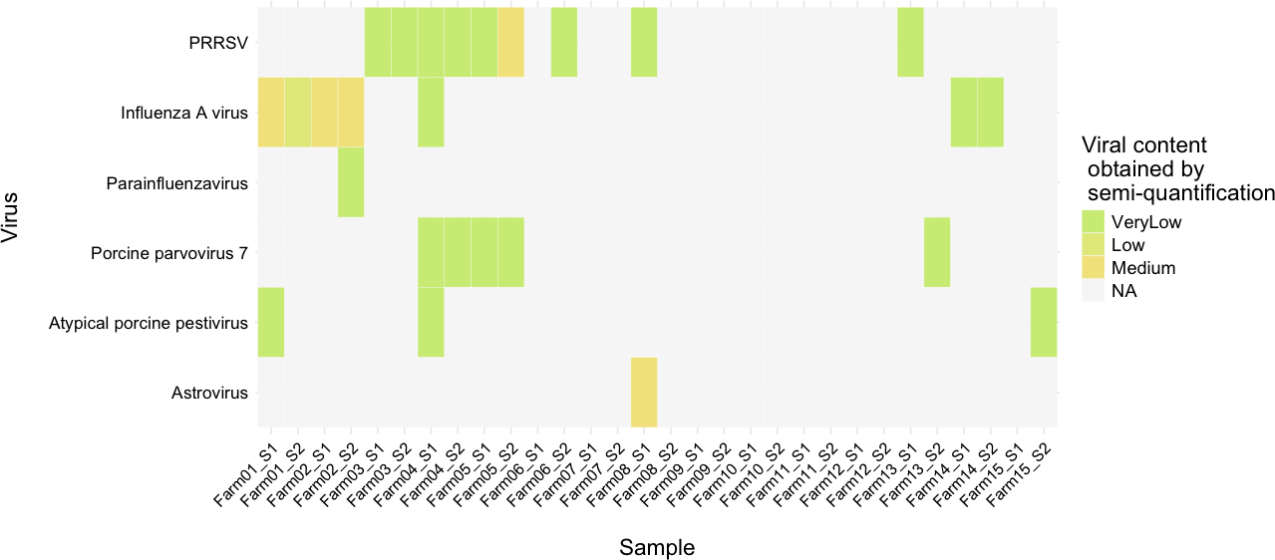
Viral loads of different viruses using the PathoSense diagnostic assay. Abundances are indicated from very high to very low based on a semi-quantification method (see Materials and Methods).

Since the co-occurrence of viruses and bacteria plays a key role in the onset of PRDC, as a next step, we performed a viral metagenomics analysis (PathoSense, Ghent, Belgium) to test the presence of viral pathogens without prior selection. The analysis was performed on the samples already used for compositional and functional microbiome analysis. The most detected viruses were Influenza A virus (swIAV) (detected in 23% of tested samples) and PRRSV (detected in 30% of tested samples). Other detected viruses (Table S1) included Astrovirus, Atypical porcine pestivirus, Porcine parvovirus 7, Parainfluenza Virus. Figure 3 depicts a semi-quantitative overview of the detected viruses in the different samples, scaled from Very Low to Very High. While this assay could also detect bacterial infections, the main focus in this case was to identify the viral infections, as more in-depth techniques for bacteria were used throughout the study. For coherence, a full list of bacteria detected through this diagnostics assay can also be found in Table S1.

### Correlation between viral and bacterial species in the tracheobronchial swabs

**Fig 4:**
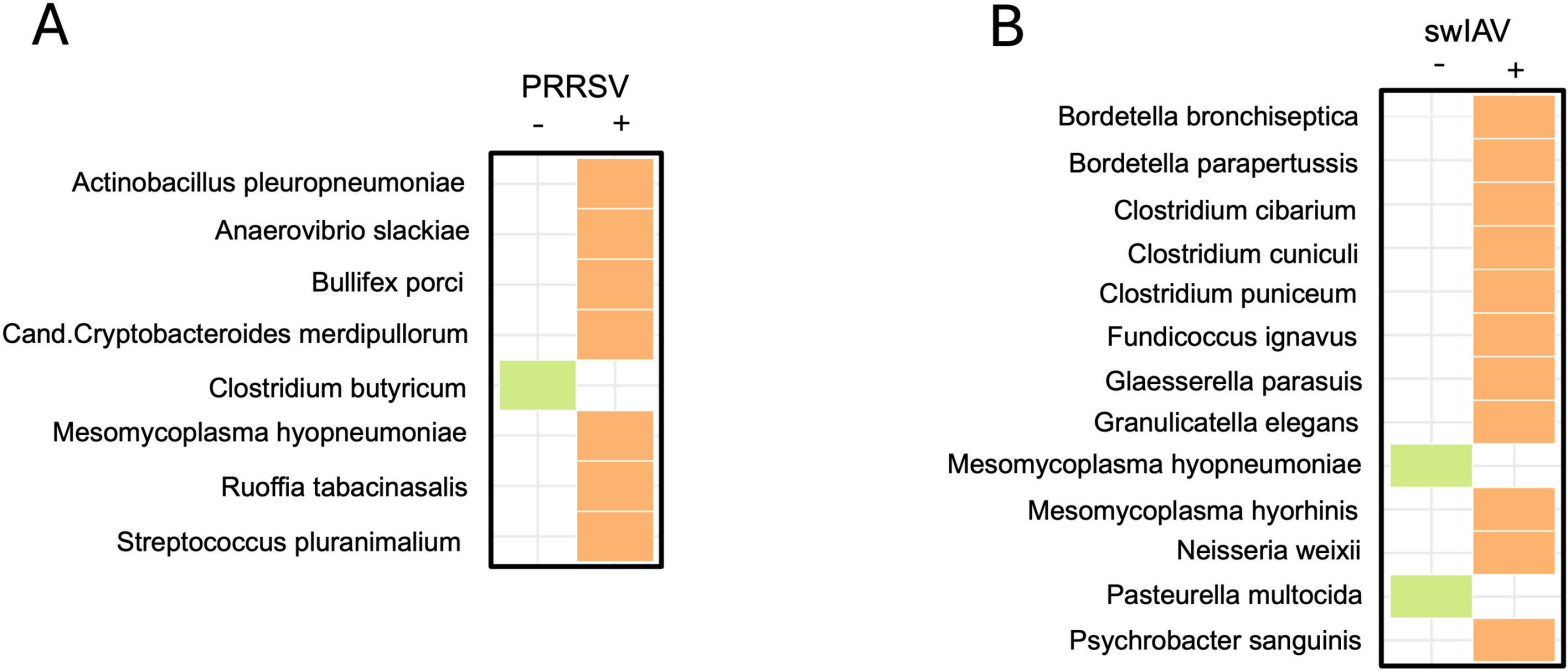
Differentially abundant bacteria between PRRSV positive and negative sample pools (**A**) and swIAV positive and negative sample pools (**B**). Differential abundance was assessed through a linear discriminant analysis (LDA).

Since bacterial and viral infections often co-exist, we next evaluated how the presence of viruses (detected via PathoSense viral metagenomics) can shape the bacterial community (detected via 16S rRNA gene sequencing) in the same collected samples. Linear Discriminant Analysis (LDA) was used to evaluate differentially abundant bacteria between PRRSV positive and negative and between swIAV positive and negative samples (Fig. 4). Interestingly, in PRRSV positive samples, *M. hyopneumoniae* was significantly more abundant (*P* = 0.036). On the other hand, in swIAV positive samples, *G. parasuis* and *M. hyorhinis* are more abundant (*P* = 0.03 and *P* = 0.006, respectively), while *M. hyopneumoniae* and *P. multocida* are less abundant (*P* = 0.001 and *P* = 0.002, respectively). Tables containing LDA scores and *P*-values for all the differentially abundant bacteria can be found in the Supplementary material (Tables S2 and S3). No significant differences in the general structure of the microbial communities were found (results not shown) comparing PRRSV positive and negative samples and swIAV positive and negative samples through beta diversity analyses.

## DISCUSSION

Despite the high importance of respiratory diseases in the pig industry, few studies have focused on in-depth characterization of the LRT microbiome. In this study, we performed a thorough characterization of the lower airways’ microbiome of a relatively large cohort of finisher pigs with acute respiratory distress employing different state-of-the-art long-read sequencing methodologies that enable not only microbial identification but also virulence factor gene profiling without prior cultivation.

A combined approach using full-length 16S rRNA gene sequencing and bacterial shotgun metagenomics revealed a bacterial profile highly consistent with respiratory disease. Four key opportunistic pathogens associated with PRDC were detected at high abundance through both techniques, that is, *M. hyopneumoniae*, *G. parasuis*, *P. multocida,* and *S. suis* (18). The rest of the microbial community was mainly constituted of genera *Streptococcus*, *Clostridium*, *Psychrobacter,* and *Rothia*. The obtained bacterial profiling is consistent with previous literature (10, 11, 13, 19), which typically describes *Mesomycoplasma*, *Glaesserella*, *Pasteurella*, and *Bordetella* as predominant in sick pigs. Conversely, studies assessing the LRT microbial composition of healthy animals reported a higher abundance of *Prevotella*, *Lactobacillus*, and *Lactococcus* (10, 11). These genera were detected in very low proportions in the present work. This finding highlights how the microbiome of animals affected by PRDC is aligned with a disease-associated composition and displaced from the health-associated, Lactobacillus-driven one.

To ensure a more comprehensive and bias-free identification, both 16S rRNA gene sequencing and bacterial shotgun metagenomics were employed. While differences can be noted between the bacterial compositions obtained through the two techniques (20) due to primer bias (21), different 16S rRNA gene copy numbers (22), or database choice, these differences ultimately provided complementary views of the microbial community. Comparative studies often highlight the superiority of shotgun sequencing for species resolution, but these studies are predominantly based on high-biomass samples (e.g., derived from the gut) (23). Given our use of low-biomass tracheobronchial mucus samples, which generally harbor a lower number of reads, we still chose a combined approach to ensure maximum reliability. We confirmed that both techniques successfully identified the main microbial patterns associated with PRDC (e.g., the high abundance of *G. parasuis* and *M. hyopneumoniae*). However, differences were detected in the low-abundance taxa. For example, shotgun sequencing identified higher *Segatella copri* in Farm 10 and *Mesomycoplasma hyorhinis* throughout all farms. Conversely, 16S sequencing identified *Clostridium lentum* in most farms, a species often undetected through shotgun sequencing. In our specific case, using both methods allowed us to capture the full taxonomic breadth in the samples. Additionally, we used long-read sequencing for both techniques, which provides an advantage over the short-read sequencing approach (4, 24), allowing for a more accurate species-level identification.

To fully understand the disease-causing potential of identified bacteria, we moved beyond simple identification. We developed a protocol for virulence factors identification that does not require bacterial culture nor the pre-selection of genes to screen for. This methodology, which can be particularly interesting for bacteria such as *M. hyopneumoniae*, that is very difficult to culture and only has a few available genome sequences in NCBI, also has the potential to be adapted for different future applications, including better assessment of strain diversity and antimicrobial resistance marker genes.

Our analysis detected several virulence factors belonging to genera *Mesomycoplasma*, *Pasteurella*, *Glaesserella*, and *Streptococcus*. Most of the identified virulence factors are not known discriminators between pathogenic or non-pathogenic strains, such as the several adhesion factors (e.g., *mhp* and *P97/P102* groups) detected in *M. hyopneumoniae* (25). At the same time, most of the detected genes are potentially involved in the pathogenesis process, such as *hlyA*, an hemolysin which has been proven to be important in the pathogenicity process of other Mycoplasmas (26), or *ptfA* in *Pasteurella*, a fimbrial gene implicated in biofilm formation (27).

Despite not yet being able to use virulence factor detection to discriminate the pathogenic potential of respiratory pathogens, this study offers a comprehensive list of VFs identified in pigs showing respiratory clinical signs, suggesting these may be more strongly associated with disease. Interestingly, we observed some correlation between VFs and bacterial abundance in specific farms. For example, *p102*, *p64*, *hly*A, and *pdhB* are particularly abundant in Farm 05, which also exhibits a high presence of *M. hyopneumoniae*. This correlation suggests these VFs might contribute to the elevated *M. hyopneumoniae* colonization rates in this farm. Future studies comparing VF profiles between healthy and diseased animals are necessary to confirm the pathogenic relevance of these factors.

To complete the picture of the PRDC, we performed viral metagenomics. Our analysis identified swIAV and PRRSV as the most prevalent viruses, in line with previous studies (28–30). Given the complex nature of PRDC, which often involves the co-occurrence of viruses and bacteria (1, 3, 30–32), we performed a differential abundance analysis between swIAV and PRRSV positive and negative samples. PRRSV-positive animals had more *M. hyopneumoniae* and *A. pleuropneumoniae*, likely due to the virus’s long presence (up to 150 days) (33) in pigs facilitating the occurrence of bacterial coinfections. Conversely, PRRSV-positive samples showed a lower level of potentially beneficial species like *Clostridium butyricum*, a known butyrate producer that has also been used as a probiotic (34, 35). This suggests that PRRSV infection can negatively impact the microbiome composition in lungs. In contrast, swIAV can only be detected for a shorter period (5–7 days post-infection), lowering the likelihood for bacterial coinfections to thrive. This may explain why some bacteria not considered main PRDC actors, such as *M. hyorhinis*, were enriched, and PRDC-associated bacteria such as *M. hyopneumoniae* and *P. multocida* were instead more abundant in swIAV-negative samples.

We believe that the present study presents valuable strengths compared to previous research in the field. First, as opposed to most previous studies (8, 36–40), we focused on LRT instead of URT. This provides a significant advantage since URT can differ in composition from the LRT (41) and may have lower sensitivity for detecting key microorganisms like *M. hyopneumoniae* (42). Moreover, we used TBS instead of lung samples, a sampling technique that can be replicated without the need for the animal to be sacrificed, offering a practical investigation tool to advance the field (4, 10, 11, 13, 43). Another strong point of this study is the use of long-read sequencing, which allows for a more reliable species-level bacterial classification.

This study presents three main limitations. First, the used samples underwent pooling. While being aware of the limitations that this process causes, it was used to improve cost-effectiveness and only after verifying that pooling can still provide reliable results and gives valuable insights on the herd level (43, 44). Additionally, pooling was consistently applied across all analyses in this study, including viral, bacterial, and functional characterization, ensuring comparability of results across different techniques. The remaining limitations relate directly to the study’s design but are contextualized by the primary aim of this work: providing a proof-of-concept study for optimizing sequencing methods and the functional exploration of the resulting microbial data. More specifically, the study lacks a comparison with healthy controls. In addition, it was not possible to quantitatively rate the symptoms of the animals, which prevented us from correlating the disease severity to the microbial profile of the animals. Future studies involving healthy controls and robust clinical scoring are required to fully address these disease dynamics.

In conclusion, this study provides valuable insights into the complex microbial environment of the LRT of pigs with PRDC. By utilizing methods to detect both viruses and bacteria, we were able to confirm the presence of viral-bacterial co-infections and identify key (*M. hyopneumoniae*, *G. parasuis*, *P. multocida*, *S. suis*) and commensal (*Streptococcus* sp., *Clostridium* sp., *Rothia* sp.) bacterial species, along with virulence factors that might exert key roles in bacterial invasion. The development of a shotgun approach suitable for low-biomass samples can have broad implications for both veterinary and human clinical research, paving the way for an improved understanding of pathogen dynamics and facilitating more accurate diagnostics for respiratory infections.

## MATERIALS AND METHODS

### Study design and sampling

Sampling was conducted between November 2023 and March 2024, coinciding with the peak period of respiratory infections. Tracheobronchial swabs were collected from pigs on 15 fattening farms located in southern Germany. Whenever animals, under monitoring of the farmer, showed acute respiratory distress, such as coughing or dyspnea, during the study period, TBS were collected as part of routine diagnostic procedures. On each farm, samples were obtained from 15 fatteners between 3 and 5 months of age, all exhibiting respiratory signs. The TBS collection has been performed by skilled swine veterinarians. To facilitate sampling a second person restrained the pig using a snare. The sampling technique followed the protocol described by Fablet (45). Briefly, a mouth gag was inserted between the jaws to allow the introduction of a sterile catheter (DCT-Nelaton Katheter; Servoprax GmbH, Germany). During inspiration, the catheter was inserted deeply into the trachea until reaching the *bifurcatio tracheae*. The onset of coughing was considered an indication of successful sampling. Following collection, the tip of the swab (4–5 cm) was cut off with scissors and transferred into a sterile 50mL sample tube containing 2 mL of phosphate buffered saline (PBS) (Roti-CELL PBS, Carl Roth GmbH + Co. KG, Karlsruhe, Germany) for subsequent transport and analysis.

### Sample processing

**Fig 5:**
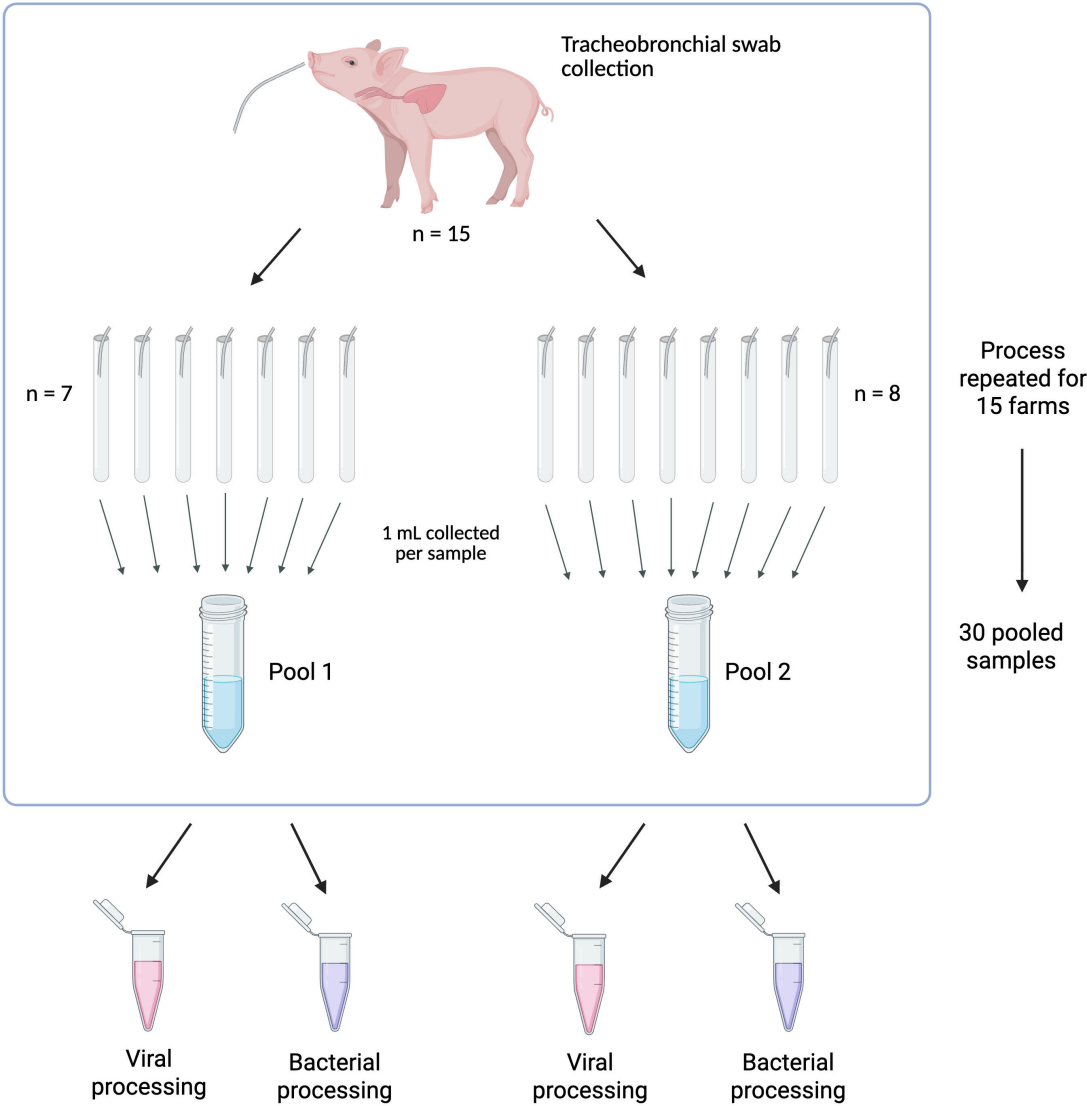
Flow chart representing the sample processing for the present study. Created in BioRender. Lebeer, S. (2025) https://BioRender.com/m8g6p08.

After collection, samples were shipped immediately to PathoSense (Ghent, Belgium) refrigerated in polystyrene boxes containing ice packs (4°C). Starting from the individual samples, two pools were formed for every farm. More specifically, for each farm, a total of 15 original samples was present. To create the pools, an aliquot of 1 mL was taken from the original sample and added to a new tube. The process was repeated for 7 of the original samples to create the first pool, and the same procedure was followed on the eight remaining samples to create the second pool. This way, two pools were generated per farm. Given the presence of 15 separate farms and 2 pools per farm, a total of 30 pools was generated. Figure 5 offers a graphical representation of the sample processing. One aliquot from each pool was processed using a patented sampler (patent WO2020260583) for initial purification from mucus and cell debris and to perform viral metagenomics. The other aliquot was used for bacterial DNA isolation for subsequent full-length 16S rRNA gene sequencing and shotgun metagenomic sequencing. These samples were either processed immediately with host depletion and DNA extraction or frozen at −80°C for later processing.

The aliquot for viral metagenomics was routinely analyzed using the PathoSense metagenomics diagnostic assay at one of PathoSense’s partner laboratories (Dierengezondheidszorg Vlaanderen, Torhout, Belgium).

### Viral characterization using the PathoSense diagnostics assay

The presence of any viruses inside the samples was detected using the PathoSense diagnostics assay, which was described previously (46–48). Briefly, samples were filtered through a 1.2 µm filter (A35621, Novolab) and subjected to a host depletion step to remove all free nucleic acids. To obtain semi-quantitative information and to ensure the good quality of the sequencing, a spike-in virus was added. Samples went through a reverse transcription step and a nucleic acid amplification step to include the detection of both DNA and RNA viruses (46, 47). Library preparation was then carried out using the SQK-RBK096 library preparation kit (ONT), and sequencing was performed on R9.4.1 flow cells (ONT). Base calling was performed using Guppy v.7.1.4. The obtained number of reads was compared to the spike-in virus to obtain a semi-quantification. Viral identification was done using an in-house pipeline developed by PathoSense and made available to partner laboratories in the cloud. The resulting number of reads was converted to a scale consisting of five levels: very low, low, medium, high, very high, as previously described (48).

### Optimization of host depletion and bacterial DNA extraction on tracheo-bronchial swabs for 16S rRNA gene sequencing

Considering the low biomass and the difficulty in obtaining sufficient amounts of bacterial DNA in TBS samples, host-depletion and DNA extraction methods were first thoroughly validated. Different procedures were evaluated, consisting of a combination of proteinase K treatment, benzonase treatment, and centrifugation at 10,000 × *g* for 5 min. A full overview of the number of bacterial reads obtained through each host depletion tested can be found in Table S4. The final obtained protocol is described hereafter. Twenty microliters of proteinase K (20 mg/mL, ProMega) was added to 250 µL of TBS sample, followed by the incubation of the sample at 55°C for 30 min. The reaction was then stopped by keeping the samples on ice for 1 min. Next, 3.5 µL of Benzonase (Merck Life Science, 25 units/µL) was added to the sample, which was then incubated at 37°C for 30 min. After the reaction, 12.5 µL of 10 nM EDTA (VWR) has been added to stop the reaction. After this host depletion step, samples were processed with the ZymoBiomics MiniPrep kit (D4300, ZymoBiomics), following the manufacturer’s instructions.

### Full-length 16S rRNA gene sequencing using nanopore sequencing

The extracted DNA has been measured using a Quantus fluorometer (Promega) and then processed for full-length 16S rRNA gene sequencing. The first step consisted of a 16S rRNA gene targeting PCR. LongAmp Taq 2X Master Mix (New England BioLabs) has been used to perform a PCR using primers 27F (5′-AGA GTT TGA TCM TGG CTC AG-3′) and 1492R (5′-ACC TTG TTA CGA CTT-3′). Reagents have been used according to the manufacturer’s protocol. PCR was carried out with 1 min at 95°C, 27 cycles of 20 s at 95°C, 30 s at 55°C for annealing, and 2 min at 65°C for elongation. A final elongation at 65°C for 5 min was also added. Sequencing of the amplicons has been performed on GridION 5 (ONT), using the Native Barcoding Kit (SQK-NBD114.24, ONT) in combination with R10.4.1 flowcells (ONT). Base calling has been performed using Guppy v.7.1.4 in the “super-accurate” setting.

Filtering was performed using chopper (https://github.com/wdecoster/chopper), retaining only reads with a Qscore > 10 and a length included between 1,300 and 1,700 base pairs. Taxonomic classification of 16S rRNA gene sequencing reads was performed using the EMU software v.3.4.5 (49) paired with the MIMt database (50).

### Bacterial functional characterization with shotgun metagenomics

The same DNA extracts used for 16S rRNA gene sequencing were also used for shotgun metagenomics sequencing. DNA was processed using the SQK-RPB114.24 kit (ONT), which allowed to correct sequencing of the low biomass samples thanks to the low input requirements. Only one slight modification from the manufacturer’s instructions was applied, as the number of PCR cycles was increased from 14 to 16. Three samples were sequenced on an R10.4.1 flow cell on GridION 5 (ONT), and the remaining 23 were sequencing on a R10.4.1 flow cell on the PromethION 2 Solo (ONT). Due to the high amount of host material in the sample, adaptive sampling (51) was performed using the pig genome as a reference (accession number GCF_000003025). Reads were base-called using Dorado v.0.7.3 in the “fast” setting first, to identify reads matching with the pig genome, and with the “super-accurate” setting after to obtain higher quality reads. Reads that did not map to the pig genome, and thus went successfully through the nanopore for sequencing, were selected and merged into a final fastq file using the catfishq tool (https://github.com/philres/catfishq). Obtained reads were classified using the Kraken software v.2.1.3 (52) paired with the kraken database k2_pluspf_20220607. Moreover, assemblies were generated using flye (https://github.com/mikolmogorov/Flye) with the metagenomic option (53). Both the obtained reads and contigs were screened for known virulence factors using abricate v1.0.1 (https://github.com/tseemann/abricate) paired with the Virulence Factors Database (vfdb full data set, http://www.mgc.ac.cn/VFs/main.htm updated to July 2024 [54]). Normalization of the virulence factors count was performed for each sample by dividing the read count of each taxon for the total bacterial read count and multiplying by 100.

### Statistical analysis

All figures were generated using R version 4.3.2. To generate heatmap figures, packages pheatmap (v. 1.0.12), ComplexHeatmap (v. 2.15.4), and ggplot2 (v. 3.5.1) were used, while to generate barplots package ggplot2 (v. 3.5.1) was used. Linear Discriminant Analysis was carried out using the microbiomeMarker package (v. 1.8.0). Only samples with an LDA log score > 2.0 were considered in the results.

## Data Availability

The sequencing data relative to the 16S rRNA gene sequencing are available in the NCBI BioProject database under accession number PRJNA1277143, while data relative to bacterial metagenomic shotgun are available under accession number PRJNA1277780.

## SUPPLEMENTAL MATERIAL

Supplemental tables spectrum.01910-25-s0001.docx Tables S1 to S4.

ASM does not own the copyrights to Supplemental Material that may be linked to, or accessed through, an article. The authors have granted ASM a non-exclusive, world-wide license to publish the Supplemental Material files. Please contact the corresponding author directly for reuse.
