# Supplementary material for: Exploring the functional microbiome of pigs within the porcine respiratory disease complex: viral-bacterial co-infections and virulence factor profiling
Source: Microbiol Spectr. 2026 Jan 29;14(3):e01910-25. doi: 10.1128/spectrum.01910-25 (PMC12955451; doi:10.1128/spectrum.01910-25)
Supplement: Supplemental tables — Tables S1 to S4. [file spectrum.01910-25-s0001.docx]

**APPENDIX**

**Table S1**. Full list of viruses and bacteria detected in each sample through the PathoSense diagnostics assay.

| **Sample** | **Detected viruses** | **Detected bacteria** |
| --- | --- | --- |
| Farm01_S1 | Influenza A virus, Atypical porcine pestivirus | *Mycoplasma hyopneumoniae, Pseudomonas sp., Glaesserella parasuis* |
| Farm01_S2 | Influenza A virus | *Mycoplasma hyopneumoniae, Pseudomonas sp., Pasteurella sp.* |
| Farm02_S1 | Influenza A virus, Hubei picorna-like virus, Wuhan insect virus | *Mesomycoplasma flocculare, Mesomycoplasma hyorhinis, Glaesserella parasuis* |
| Farm02_S2 | Influenza A virus, Parainfluenza virus | *Mesomycoplasma flocculare, Mesomycoplasma hyorhinis, Glaesserella parasuis* |
| Farm03_S1 | PRRSV | *Mesomycoplasma hyopneumoniae, Pasteurella sp.* |
| Farm03_S2 | PRRSV | *Mesomycoplasma hyopneumoniae, Mesomycoplasma flocculare, Glaesserella parasuis* |
| Farm04_S1 | PRRSV, Influenza A virus, Atypical porcine pestivirus, Porcine parvovirus 7 | *Mesomycoplasma hyopneumoniae* |
| Farm04_S2 | PRRSV, Porcine parvovirus 7 | *Mesomycoplasma hyopneumoniae* |
| Farm05_S1 | PRRSV, Porcine parvovirus 7 | *Mesomycoplasma hyopneumoniae* |
| Farm05_S2 | PRRSV, Porcine parvovirus 7 | *Mesomycoplasma hyopneumoniae, Pasteurella sp.* |
| Farm06_S1 | None | *Mesomycoplasma hyopneumoniae, Mycoplasmopsis felis, Glaesserella parasuis* |
| Farm06_S2 | PRRSV | *Mesomycoplasma hyopneumoniae, Mycoplasmopsis felis, Glaesserella parasuis* |
| Farm07_S1 | None | *Mesomycoplasma hyopneumoniae, Pasteurella sp.* |
| Farm07_S2 | None | *Mesomycoplasma hyopneumoniae, Mesomycoplasma hyorhinis, Pasteurella sp.* |
| Farm08_S1 | PRRSV, Astrovirus | *Mesomycoplasma hyopneumoniae, Mesomycoplasma hyorhinis, Pasteurella sp.* |
| Farm08_S2 | None | *Mesomycoplasma hyopneumoniae; Mesomycoplasma hyorhinis* |
| Farm09_S1 | Picobirnavirus, Porcine parvovirus 7, Rotavirus B, Porcine picobirnavirus, Torque teno sus virus | *Mesomycoplasma hyopneumoniae, Pseudomonas sp. P4(2010), Achromobacter sp., Pasteurella sp.* |
| Farm09_S2 | Picobirnavirus, Porcine parvovirus 7, Porcine picobirnavirus | *Mesomycoplasma hyopneumoniae, Pseudomonas sp., Achromobacter sp., Pasteurella sp.* |
| Farm10_S1 | None | *Mycoplasma hyopneumoniae, Pasteurella sp.* |
| Farm10_S2 | None | *Mycoplasma hyopneumoniae, Pasteurella sp., Glaesserella sp.* |
| Farm11_S1 | None | *Mycoplasma hyopneumoniae, Glaesserella sp.* |
| Farm11_S2 | None | *Mycoplasma hyopneumoniae, Bordetella sp., Glaesserella sp.* |
| Farm12_S1 | None | *Mycoplasma hyopneumoniae* |
| Farm12_S2 | None | *Mycoplasma hyopneumoniae, Pasteurella sp.* |
| Farm13_S1 | PRRSV | *Mycoplasma hyopneumoniae, Pseudomonas sp., Shewanella sp., Pasteurella sp.* |
| Farm13_S2 | Porcine parvovirus 7 | *Mycoplasma hyopneumoniae, Pseudomonas fragi, Shewanella sp., Pasteurella sp.* |
| Farm14_S1 | Influenza A virus | *Mycoplasma flocculare, Moraxella sp.* |
| Farm14_S2 | Influenza A virus | *Mycoplasma flocculare* |
| Farm15_S1 | None | *Pasteurella sp., Pseudomonas sp., Mesomycoplasma hyopneumoniae* |
| Farm15_S2 | Atypical porcine pestivirus | *Pasteurella sp., Pseudomonas sp., Mesomycoplasma hyopneumoniae* |

**Table S2**. LDA scores and p-adjusted values for each differentially abundant species from the LDA analysis comparing PRRSV positive and negative samples.

| Differentially abundant species | Enriched group | LDA score | p-adjusted value |
| --- | --- | --- | --- |
| Clostridium butyricum | PRRSV_Negative | 4.074 | 0.036 |
| Mesomycoplasma hyopneumoniae | PRRSV_Positive | 5.372 | 0.036 |
| Anaerovibrio slackiae | PRRSV_Positive | 3.802 | 0.211 |
| Actinobacillus pleuropneumoniae | PRRSV_Positive | 3.780 | 0.009 |
| Streptococcus pluranimalium | PRRSV_Positive | 3.283 | 0.040 |
| Ruoffia tabacinasalis | PRRSV_Positive | 3.276 | 0.023 |
| Bullifex porci | PRRSV_Positive | 2.702 | 0.047 |
| Candidatus Cryptobacteroides merdipullorum | PRRSV_Positive | 2.670 | 0.024 |

**Table S3**. LDA scores and p-adjusted values for each differentially abundant species from the LDA analysis comparing swIAV positive and negative samples.

| Differentially abundant species | Enriched group | LDA score | p-adjusted value |
| --- | --- | --- | --- |
| Mesomycoplasma hyopneumoniae | swIAV_Negative | 5.364 | 0.001 |
| Pasteurella multocida | swIAV_Negative | 5.083 | 0.002 |
| Glaesserella parasuis | swIAV_Positive | 4.838 | 0.03 |
| Clostridium cuniculi | swIAV_Positive | 4.425 | 0.036 |
| Mesomycoplasma hyorhinis | swIAV_Positive | 4.252 | 0.006 |
| Psychrobacter sanguinis | swIAV_Positive | 4.066 | 0.019 |
| Clostridium puniceum | swIAV_Positive | 3.752 | 0.027 |
| Clostridium cibarium | swIAV_Positive | 3.544 | 0.005 |
| Bordetella bronchiseptica | swIAV_Positive | 3.521 | 0.013 |
| Bordetella parapertussis | swIAV_Positive | 3.518 | 0.013 |
| Granulicatella elegans | swIAV_Positive | 3.473 | 0.043 |
| Fundicoccus ignavus | swIAV_Positive | 2.884 | 0.013 |
| Neisseria weixii | swIAV_Positive | 2.780 | 0.043 |

**Table S4**. Total number of sequenced reads, number and percentage reads mapped to a bacterial database. The different tested treatments are abbreviated as follows: PBW = Proteinase + Benzonase; PBP = Proteinase + Benzonase, pellet only; PBS = Proteinase + Benzonase, supernatant only; BW = Benzonase; BP = Benzonase, pellet only; BS = Benzonase, supernatant only; P = proteinase K; M = MiniPrep only, CB = Centrifugation + Benzonase; C = Centrifugation.

| **Host depletion method** | **Total reads** | **Mapped reads** | **% mapped reads** |
| --- | --- | --- | --- |
| **PBW** | 88579 | 39448 | 44.53 |
| **PBP** | 84196 | 48259 | 57.32 |
| **PBS** | 714 | 61 | 8.48 |
| **BW** | 27459 | 7188 | 26.18 |
| **BP** | 33554 | 6568 | 19.57 |
| **BS** | 11219 | 3104 | 27.66 |
| **P** | 25134 | 2561 | 10.19 |
| **M** | 33816 | 3121 | 9.23 |
| **CB** | 33821 | 4665 | 13.79 |
| **C** | 8461 | 562 | 6.64 |
